# Assessing the Temporal Organization of Walking Variability: A Systematic Review and Consensus Guidelines on Detrended Fluctuation Analysis

**DOI:** 10.3389/fphys.2020.00562

**Published:** 2020-06-23

**Authors:** Deepak K. Ravi, Vivien Marmelat, William R. Taylor, Karl M. Newell, Nick Stergiou, Navrag B. Singh

**Affiliations:** ^1^Institute for Biomechanics, ETH Zurich, Zurich, Switzerland; ^2^Department of Biomechanics and Center for Research in Human Movement Variability, University of Nebraska at Omaha, Omaha, NE, United States; ^3^Department of Kinesiology, University of Georgia, Athens, GA, United States

**Keywords:** fractal fluctuations, fractal temporal structures, gait variability, stride interval variability, detrended fluctuation analysis, long-range correlations, aging, Parkinson disease

## Abstract

Human physiological signals are inherently rhythmic and have a hallmark feature in that even distant intrasignal measurements are related to each other. This relationship is termed long-range correlation and has been recognized as an indicator of the optimal state of the observed physiological systems, among which the locomotor system. Loss of long-range correlations has been found as a result of aging as well as disease, which can be evaluated with detrended fluctuation analysis (DFA). Recently, DFA and the scaling exponent α have been employed for understanding the degeneration of temporal regulation of human walking biorhythms in, for example, Parkinson disease (PD). However, heterogeneous evidence on scaling exponent α values reported in the literature across different population groups has put into question what constitutes a healthy physiological pattern. Therefore, the purpose of this systematic review was to investigate the functional thresholds of scaling exponent α in young vs. older adults, as well as between patients with PD and age-matched asymptomatic controls. Aging and PD exhibited a negative effect size (i.e., led to decreased long-range correlations) of −0.20 and −0.53, respectively. Our meta-analysis based on 14 studies provides evidence that a mean scaling exponent α threshold of 0.86 [2 standard error (0.76, 0.96)] is able to optimally discriminate temporal organization of stride interval between young and old, whereas 0.82 (0.72, 0.92) differentiates patients with PD and age-matched asymptomatic controls. The optimal thresholds presented in this review together with the consensus guidelines for using DFA might allow a more sensitive and reliable application of this metric for understanding human walking physiology than has been achieved to date.

## Introduction

Walking is regulated and coordinated through spinal and supraspinal sensorimotor networks allowing humans to adapt to both intrinsic and extrinsic challenges and perturbations (Dingwell et al., 2010; Takakusaki, 2017). During this regulation to achieve stable walking, natural fluctuations are present between strides in both the temporal (e.g., stride interval) and spatial (e.g., step width) domains (Hausdorff, 2005, 2007). These stride-to-stride fluctuations during walking have been characterized using approaches that evaluate not only the overall magnitude (Hausdorff et al., 2001; Konig et al., 2016a), but also the temporal organization of this walking variability (Hausdorff, 2007; Stergiou and Decker, 2011). Parameters that are used to evaluate the magnitude of walking variability are highly useful [e.g., standard deviation (SD) values have shown potential diagnostic and prognostic applications in a number of pathologies; (Hausdorff, 2005, 2009; Stergiou and Decker, 2011; Konig et al., 2016a,b; Ravi et al., 2019)], but do not provide information on how walking behavior evolves over time (West, 1990; Lipsitz and Goldberger, 1992). Nevertheless, a large number of studies that have evaluated the temporal organization of walking variability support the hypothesis that stride-to-stride fluctuations in the duration of gait cycles (synonymous with the stride interval, also sometimes termed “stride time”) exhibit long-range correlations; that is, at any instance of walking, one-stride interval is correlated to stride intervals at relatively distant time points (Hausdorff et al., 1995; Griffin et al., 2000; Hu et al., 2004). While these long-range correlations are independent of the size of the window, or “scale” of observation, the decay of these correlations can be modeled as a power law (West and Griffin, 1999). Furthermore, the rate of decay can be quantified using scaling exponents that analytically characterize the presence of a temporal organization within an observed time series (Peng et al., 1995a; West and Griffin, 1999).

Detrended fluctuation analysis (DFA) allows a characterization of the nature of long-range correlations in the stride interval of walking (Pierrynowski et al., 2005; Bashan et al., 2008; Damouras et al., 2010; Choi et al., 2015; Marmelat et al., 2018). The scaling exponent α calculated using this method is robust against non-stationarity, artifacts, and related missing data (Chen et al., 2002; Ma et al., 2010). Importantly, however, it allows the temporal organization of walking variability to be characterized accurately within relatively short time series consisting of only a few hundred or more strides (Delignieres et al., 2006; Almurad and Delignieres, 2016; Kuznetsov and Rhea, 2017). Scaling exponent α also exhibits good intraday and interday reliability (Pierrynowski et al., 2005; Choi et al., 2015). As a result, this method has become favored compared to other approaches for evaluating temporal organization of walking variability. Notably, the scaling exponent α is not significantly correlated with the magnitude of walking variability, and the two approaches might therefore elucidate different physiological control mechanisms (Hausdorff, 2007, 2009; Uchitomi et al., 2013; Ota et al., 2014). In the absence of long-range correlations, such as a fully random time series, the scaling exponent α approaches the value of 0.5. Values lower than 0.5 indicate “antipersistent” behavior (e.g., large stride-to-stride fluctuations tend to be followed by smaller fluctuations, and *vice versa*). While antipersistent behavior has been reported in cases of healthy cardiac dynamics (Bartsch et al., 2005), it is not commonly observed in natural or pathological walking patterns. Values higher than 0.5 indicate “persistent” behavior (e.g., large stride-to-stride fluctuations tend to be followed by larger fluctuations, and small stride-to-stride fluctuations by smaller). Previous research indicates that stride intervals during walking exhibit an organized behavior and hence generally present persistent long-range correlations, although the exact length of correlations and the mechanisms behind this behavior continue to be discussed (Hausdorff et al., 1995, 1996; West and Scafetta, 2003; Hausdorff, 2007).

Some argue that the presence of long-range correlations is indicative of deterministic regulation of stride intervals, hence reflecting stable but flexible walking behavior (ability to adapt to changing task demands), which is observed in healthy individuals (Stergiou and Decker, 2011; Manor and Lipsitz, 2013; Terrier and Deriaz, 2013; Chien et al., 2015; Warlop et al., 2016; Ducharme et al., 2019). As a result, a scaling exponent α of ~1 has traditionally been interpreted to represent healthy movement patterns (Hausdorff, 2007, 2009; Gow et al., 2017). Movement disorders due to aging and neurological diseases, for example, Parkinson disease [PD (Frenkel-Toledo et al., 2005; Hausdorff, 2009; Marmelat et al., 2018), but also Huntington's disease (Hausdorff et al., 1997), as well as cognitive decline (Lamoth et al., 2011)], are associated with a loss of persistence (Damouras et al., 2010; Stergiou and Decker, 2011; Ota et al., 2014; Li et al., 2019) and hence lower scaling exponent α values (nearer to 0.5). The implication is that neural pathologies might adversely influence mechanisms that regulate the nature of long-range correlations in walking. Because of their functional significance, it is not surprising that such long-range power-law correlations have also been observed in other physiological processes including cardiac, respiratory, and neural rhythms, as well as their deterioration with pathologies (Peng et al., 1995a; Bartsch et al., 2005; Ivanov et al., 2009; Werner, 2010). However, the literature also suggests that differences in the values of scaling exponent α between cohorts (e.g., young vs. older adults, or between patients with PD and age-matched asymptomatic controls) are often small (overall cohort differences in scaling exponent α = ~0.05). In this respect, a clear characterization of the relative values of α between populations with differing neurological statuses is needed. In addition, the interpretation of the scaling exponent α in human movement research remains ambiguous as considerable diversity in α values has been reported across similar population groups (Hausdorff et al., 1997; Kobsar et al., 2014; Kosse et al., 2016; Dotov et al., 2017; Marmelat et al., 2018). Importantly, it remains unknown whether the implementation of DFA could be critical in driving such inconsistency. As a result, the prognostic value of the scaling exponent α for characterizing differences in movement patterns, not only between healthy and pathology, but also due to aging, remains to be established.

Consequently, the following questions arise: (1) Can the value of the scaling exponent α truly reflect the “health” of a group and/or an individual? (2) If so, what are the optimal thresholds of the scaling exponent α that distinguish young from asymptomatic elderly and from pathological walking patterns? And (3) what methodological techniques are required in order to provide reliable scaling exponent α estimates? With the aim to provide science-based evidence for establishing the usage of scaling exponent α in clinical settings, this study directly addresses these issues by undertaking a systematic review and meta-analysis of the literature to understand the thresholds in scaling exponent α for discriminating young vs. older adults, as well as between patients with PD [as a neuromuscular pathology of clinical interest with a relatively well-studied population (Hausdorff, 2009; Moon et al., 2016)] and age-matched asymptomatic controls.

## Methods

### Publication Search and Selection

A systematic search of the literature was conducted in April 2019 using the databases PubMed, ISI Web of Knowledge, EBSCO, and EMBASE for peer-reviewed articles. Our aim was to comprehensively identify studies reporting the effects of aging and PD on the temporal organization of walking variability. The inclusion criteria for the studies were as follows: (1) **P**opulation (without **I**ntervention) and **C**omparison—cohort of old vs. younger adults, or a cohort of patients with PD vs. age-matched asymptomatic controls, (2) **O**utcome—stride interval dynamics expressed by DFA scaling exponent α, and (3) Task—walking on a treadmill or overground at a comfortable or self-selected walking speed. The search string was made specific to each database and was constructed using Boolean operators so that an “AND” combination of terms specified the task (e.g., walk^*^), outcome (e.g., DFA), and population (e.g., old^*^). Within these categories, synonyms as well as additional terms were specified using the “OR” operator, while a “NOT” condition was used to exclude publications involving, for example, animals, children, and so on. The search was additionally limited to original research articles published after the year 1980. The complete search string is provided in the electronic Supplementary Material, Supplementary Methods 1. The review protocol was registered in PROSPERO (registration no. CRD42018110108).

**Figure 1:**
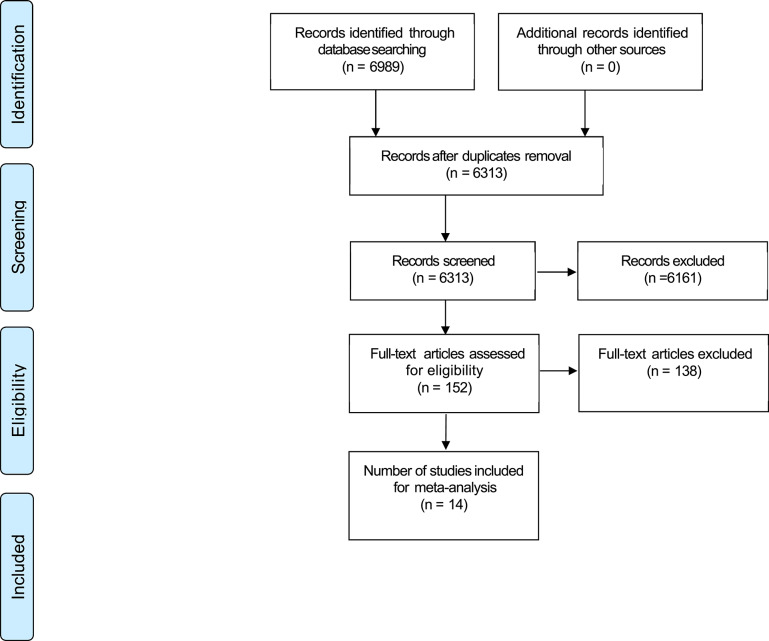
PRISMA flowchart.

One of the authors (D.K.R.) performed the literature search and screened the studies at each stage of the review. The study was performed according to the guidelines provided by the Preferred Reporting Items for Systematic Reviews and Meta-Analysis (PRISMA flowchart, Figure 1). The literature search identified 6,989 potentially relevant articles. After the removal of duplicates (*n* = 676) and articles rejected based on title or abstract (*n* = 6,161), 152 articles were included for full text screening. During this process, 138 articles were further excluded because of our inclusion/exclusion criteria (see Supplementary Material, Supplementary Methods 2). Finally, 14 studies were included in the review and meta-analysis (Table 1). Screening of all articles was performed within the EPPI-Reviewer 4 software (EPPI-Centre, UCL Institute of Education, University of London, London, UK).

**Table 1:** Characteristics of studies included in the review.

**Study ID**	**Author**	**Date**	**Technology**	**Sampling** **rate** **(Hz)**	**Experimental** **setup**	**Trial** **duration (min)**	**Cohort**	**Sample** **size**	**Age (y)**	**Box** **size**	***N***	**PD** state^*^	**PD** score^**^
1	Marmelat	2018	Footswitches	1,942	Overground	15	Healthy young	15	23 (1.69)	10–*N*/8	512		
1	Marmelat	2018	Footswitches	1,942	Overground	15	Healthy old	14	67.71 (5.92)	11–*N*/8	512		
1	Marmelat	2018	Footswitches	1,942	Overground	15	Patients with PD	13	71.31 (6.12)	12–*N*/8	512	ON	1.64 (0.69)
2	Dotov	2017	IMU	128	Overground	3	Patients with PD	19	60.8 (10.6)	4–*N*/4	120–195	ON	2 (1–3)
2	Dotov	2017	IMU	128	Overground	3	Healthy old	19	60.8 (10.6)	4–*N*/4	120–195		
3	Stout	2016	Motion capture	200	Treadmill	15	Healthy young	10	25.2 (1.5)	16–*N*/9	652		
3	Stout	2016	Motion capture	200	Treadmill	15	Healthy old	10	59.6 (10.7)	16–*N*/9	719		
4	Kosse	2016	iPod touch	88–92	Overground	3	Healthy young	29	28 (7)	17–*N*/7	—		
4	Kosse	2016	iPod touch	88–92	Overground	3	Healthy old	30	62 (8)	17–*N*/7	—		
5	Chien	2015	Motion capture	100	Treadmill	5	Healthy young	10	27 (4)	4–*N*/4	200		
5	Chien	2015	Motion capture	100	Treadmill	5	Healthy old	7	70 (10)	4–*N*/4	200		
6	Ota	2014	Foot switches	100	Overground	2–4	Patients with PD	45	69.8 (8.2)	10–*N*/2	154	ON	1–3
6	Ota	2014	Foot switches	100	Overground	2–4	Healthy old	17	70.2 (2.8)	10–*N*/2	154		
7	Kobsar	2014	IMU	100	Overground	10	Healthy young	41	24 (3)	4–*N*/2	1,000		
7	Kobsar	2014	IMU	100	Overground	10	Healthy old	41	76 (5)	4–*N*/2	1,024		
8	Kaipust	2013	Motion capture	100	Treadmill	6	Healthy young	27	25.7 (3.0)	—	151		
8	Kaipust	2013	Motion capture	100	Treadmill	6	Healthy old	27	71.4 (4.4)	—	151		
9	Bartsch	2007	Foot switches	100	Overground	2	Healthy old	24	64.3 (1.3)	*N*/10 s length	~120		
9	Bartsch	2007	Foot switches	100	Overground	2	Patients with PD	29	67 (1.3)	*N*/10 s length	~120	ON	—
10	Warlop	2006	IMU	512	Overground	10	Patients with PD	20	65.3 (9.6)	10–*N*/2	512	ON	2 (1–3)
10	Warlop	2006	IMU	512	Overground	10	Healthy old	15	60.1 (13.3)	10–*N*/2	512		
11	Toledo	2005	Foot switches	100	Overground	2	Patients with PD	36	61.2 (9.0)	—	—	—	
11	Toledo	2005	Foot switches	100	Overground	2	Healthy old	30	57.7 (7.0)	—	—		
12	Malatesta	2003	Foot switches	100	Treadmill	6	Healthy old	10	65.3 (2.5)	10–20	—		
12	Malatesta	2003	Foot switches	100	Treadmill	6	Healthy young	10	24.6 (2.6)	10–20	—		
13	Hausdorff	2000	Foot switches	300	Overground	5	Patients with PD	15	47 (29–71)	10–20	—	ON	1–4
13	Hausdorff	2000	Foot switches	300	Overground	5	Healthy old	16	67 (44–80)	10–20	—		
14	Hausdorff	1997	Foot switches	300	Overground	6	Healthy old	10	75.7 (3.2)	10–20	316		
14	Hausdorff	1997	Foot switches	300	Overground	6	Healthy young	22	24.6 (1.9)	10–20	315		

*Reported as mean (standard deviation) or median (lower limit–higher limit)*.

*“—” indicates not reported*.

* *Medication state*.

** *Hoehn and Yahr scale*.

### Study Quality Assessment

The MINORS tool (Slim et al., 2003) was used for assessing the risk of bias for the included studies. Two authors (D.K.R. and N.B.S.) independently assessed each study as having a high, unclear, or low risk of bias (scored from 0 to 2, respectively, with a maximum score of 18) on all the items included in the original checklist (except three items: “Unbiased assessment of the study endpoint,” “Follow-up period appropriate to the aim of the study,” and “Loss to follow-up less than 5%” were considered not relevant and hence excluded). Disagreements were resolved through consensus, resulting in an agreed risk-of-bias score (Table S1).

### Optimal Thresholds

In order to assess the effect of age and pathology on the scaling exponent α of the stride interval of walking and later to determine the thresholds between populations, means and SDs of the scaling exponent α were extracted from each manuscript. In studies in which the standard error of the mean or 95% confidence intervals (CIs) were presented instead of SD, these values were translated into SD according to the recommendations provided by Cochrane (Higgins and Green, 2011). An effect size (ES; the difference between the means of the two groups over the pooled SD), corrected for sample size to provide Hedges' *g* (Lipsey and Wilson, 2001), was evaluated for each study to express the difference between cohorts in a standardized manner. The results from all studies were then combined by calculating a pooled ES using the standard error as a weighting factor (in order to minimize the risk of overestimation). Heterogeneity was assessed using Cochran's *Q* and *I*^2^ statistics. In order to determine the upper threshold discriminating healthy asymptomatic from pathological gait, all studies that matched the sign of the pooled ES were selected (Konig et al., 2016a; Ravi et al., 2019). We then applied a mixed-effects binary logistic regression analysis (BLR) in order to assess how the scaling exponent α differentiates the temporal organization of walking variability of young vs. older adults and patients with PD vs. age-matched asymptomatic controls. Briefly, we modeled the log odds of the binary outcome (0 for healthy, 1 for pathological) as a linear combination of the predictor variable (scaling exponent α) and the study index (random effects). The logistic model is given by:

(1) $$\begin{array}{r} log\left(\frac{p_{i}}{1-p_{i}}\right)=\;B_{0}+\;B_{1}*x_{i}+U_{i} \end{array}$$

where *p*_*i*_ is the probability that the observation belonged to a particular cohort given the predictor variable, *x*_*i*_ (scaling exponent α), and the study index, *U*_*i*_; *B*_0_, and *B*_1_, are coefficient estimates of the regression model estimated using maximum likelihood. The quality of the classification of our model was assessed using the receiver operating characteristic (ROC) curve obtained by representing sensitivity vs. specificity for all possible values of the cutoff point between pathology and healthy. Here, sensitivity (true-positive rate) was defined as the probability of correctly classifying an outcome as pathological, whereas specificity (true-negative rate) was the probability of correctly classifying an outcome as healthy. We then identified the optimum cutoff probability point *P*_*opt*_, as the point minimizing the Euclidean distance between the ROC curve and the (1,1) coordinate on the ROC plane (Zweig and Campbell, 1993) and used an inverse binary logistic regression function (2) in order to assess the optimal threshold value *x*_*opt*_ given by:

(2) $$\begin{array}{r} x_{opt}=\;\frac{\left(\log\frac{P_{opt}}{\left(1-P_{opt}\right)}\;-{\;B}_{0}\right)}{B_{1}} \end{array}$$

The standard error (*SE*) of the estimated thresholds was evaluated using the delta method involving a first-order Taylor approximation (Venables and Ripley, 2010), as given by:

(3) $$\begin{array}{r} SE\left(x_{opt}\right)=\;\sqrt{\left[\begin{array}{cc} a & b \end{array}\right]*\left[\begin{array}{cc} var\left(B_{0}\right) & cov\left(B_{0},B_{1}\right)\\ cov\left(B_{0},B_{1}\right) & var\left(B_{1}\right) \end{array}\right]*\left[\begin{array}{c} a\\ b \end{array}\right]} \end{array}$$

where

$$\begin{array}{l} \;a=\frac{\partial x_{opt}}{\partial B_{0}}=\;-\frac{1}{B_{1}}\\ b=\;\frac{\partial x_{opt}}{\partial B_{1}}=\;-\frac{x_{opt}}{B_{1}} \end{array}$$

All analyses were conducted in MATLAB 2016b (The MathWorks Inc., Natick, MA, USA) and R (v3.4.1; The R Foundation for Statistical Computing, Vienna, Austria).

### Methodological Choices and Other Study Characteristics

In addition to means and SDs of the scaling exponent α, we extracted the following data from each manuscript: technology used to collect the three-dimensional (3D) kinematics data, sampling rate, experimental setup (overground vs. treadmill), trial duration, length of time series, sample size, window sizes, magnitude of stride interval variability, and PD-related demographics (disease stage, medication). The information extracted from the articles is presented in the Table 1.

## Results

Fourteen studies were identified as eligible for inclusion in this review. Of these 14, seven investigated the effects of aging on the scaling exponent α of the stride interval of walking, six investigated the effects of PD, and one study investigated both the effects of aging and PD. Ten studies used overground walking for their experimental protocol, and four used a treadmill-based walking protocol. The walking patterns were assessed using footswitches (seven studies), inertial measurement units (three studies), motion capture systems (three studies), and off-the-shelf smart devices (one study). Most importantly, only four studies included 512 strides or more in their analysis, which is what has previously been reported as required for the reliable assessment of the scaling exponent α using DFA (Delignieres et al., 2006). Study averages for all population groups indicate persistent long-range correlations (i.e., 0.5 < α < 1) in the stride interval of walking. The articles included for the meta-analysis presented varying risk of bias and were of mixed methodological quality. Mean quality score was 13.07 ± 1.28 (range, 11–15) against a maximum score of 18. The summary of the methodological score for each question and studies is provided in Table S1.

### Effect of Aging on Scaling Exponent α

**Figure 2:**
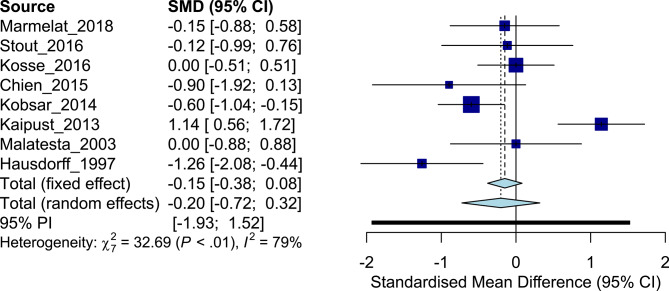
Forest plot: young adults vs. older adults.

The eight studies addressing effects of aging included a total of 149 older adults [cohort average age, 68.5 ± 6.0 years (mean ± SD)] and 164 young adults (25.3 ± 1.6 years). Meta-analysis of the studies produced an overall ES of −0.20, with older adults having generally lower levels of the scaling exponent α compared to their younger counterparts [cohort average α old, 0.77 (0.15) vs. young: 0.79 (0.13)] and individual ES ranging from −1.26, to zero, to 1.14 (Figure 2). Based on the studies that exhibited an ES aligned with the overall ES [*n* = 5; average α old, 0.77 (0.12) vs. young: 0.84 (0.09); Table 2], BLR revealed an area under the curve (AUC) of 0.76, a sensitivity of 0.80, and a specificity of 0.60 (Figures S1, S2). Here, inverse regression revealed the optimal threshold (−2 SE, +2 SE) of physiological scaling exponent α of the stride interval of walking to be 0.86 (0.76, 0.96). Substantial inconsistency was observed across studies (*I*^2^ = 79%, *Q* = 32.69). Compared to the scaling exponent α, the magnitude of variability of the stride interval also had a low mean ES (Hedges' *g*: 0.38, indicating increased levels of variability in older subjects, Table 2).

**Table 2:** Effect size comparisons: young adults vs. older adults.

**References**	**DFA scaling exponent α of stride interval Mean (SD)**		**Hedges' *g* effect size**	**Stride interval variability in % CV Mean (SD)**		**Hedges' *g* effect size**
	**Young adults**	**Older adults**		**Young adults**	**Older adults**	
Marmelat et al. (2018)	0.89 (0.09)	0.87 (0.16)	−0.15	1.76 (0.46)	1.71 (0.49)	−0.10
Stout et al. (2016)	0.86 (0.06)	0.85 (0.1)	−0.12	—	—	—
Kosse et al. (2016)	0.7 (0.17)	0.7 (0.15)	0.00	3.3 (1.45)	3.4 (1.94)	0.06
Chien et al. (2015)	0.76 (0.07)	0.69 (0.08)	−0.90	2.02 (0.72)	2.93 (1.49)	0.79
Kobsar et al. (2014)	0.83 (0.1)	0.76 (0.13)	−0.60	2.15 (0.71)	2.60 (0.91)	0.55
Kaipust et al. (2013)	0.6 (0.2)	0.85 (0.23)	1.14	—	—	—
Hausdorff et al. (2000)	0.78 (0.17)	0.78 (0.22)	0.00	1.40 (0.30)	1.93 (0.39)	1.46
Hausdorff et al. (1997)	0.87 (0.15)	0.68 (0.14)	−1.26	1.96 (0.4)	2.0 (0.7)	0.08

“*—” indicates not reported*.

### Effect of PD on Scaling Exponent α

**Figure 3:**
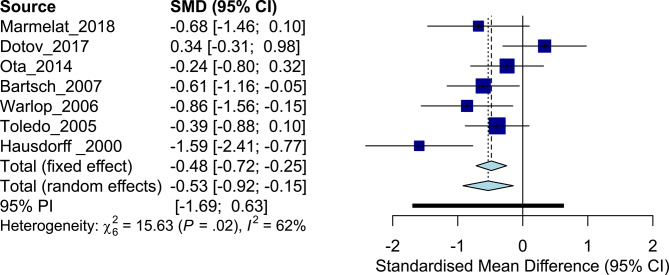
Forest plot: patients with Parkinson disease vs. age-matched asymptomatic controls.

The systematic search revealed seven studies that included a total of 177 patients with PD (cohort average age, 63.2 ± 8.2 years) and 135 healthy controls (64.0 ± 5.0 years). All the studies (except one study that did not report values) tested patients in the ON-medication state. Disease severity was most commonly evaluated using the Hoehn and Yahr scale (Hoehn and Yahr, 1967) (four studies recruited patients in the range 1–3 and one study recruited between 1 and 4). The overall ES was −0.53, whereas the individual ES ranged from [0.34 to −1.59] (Figure 3), with considerable inconsistency across trial results (*I*^2^ = 62%, *Q* = 15.63). Patients with PD generally exhibited less persistent fluctuations in stride interval of walking compared to age-matched asymptomatic controls [cohort average α patients with PD, 0.74 (0.14) vs. healthy asymptomatic, 0.81 (0.14)]. Binary logistic regression analysis based on the studies that exhibited an ES aligned with the overall ES [*n* = 6; average α patients with PD, 0.75 (0.13) vs. healthy asymptomatic, 0.83 (0.13); Table 3] revealed an AUC of 0.79, a sensitivity of 0.83, and a specificity of 0.67 (Figures S3, S4). Inverse regression revealed the higher bound of physiological scaling exponent α of stride interval of walking to be 0.82 (0.72, 0.92). The magnitude of variability of stride interval had a large mean Hedges' *g* (1.28, Table 3) compared to the scaling exponent α.

**Table 3:** Effect size comparisons: patients with Parkinson disease vs. age-matched asymptomatic controls.

**Refernces**	**DFA scaling exponent α of stride interval Mean (SD)**		**Hedges' *g* effect size**	**Stride interval variability in % CV Mean (SD)**		**Hedges' *g* effect size**
	**Healthy controls**	**Patients with PD**		**Healthy controls**	**Patients with PD**	
Marmelat et al. (2018)	0.87 (0.16)	0.77 (0.12)	−0.68	1.71 (0.49)	2.20 (0.85)	0.69
Dotov et al. (2017)	0.64 (0.17)	0.7 (0.18)	0.34	1.06 (0.30)	1.40 (0.70)	0.62
Ota et al. (2014)	0.83 (0.24)	0.78 (0.19)	−0.24	—	—	—
Bartsch et al. (2007)	0.92 (0.15)	0.84 (0.11)	−0.61	—	—	—
Warlop et al. (2006)	0.78 (0.07)	0.66 (0.17)	−0.86	1.91 (0.54)	2.88 (1.34)	0.88
Frenkel-Toledo et al. (2005)	0.69 (0.12)	0.64 (0.13)	−0.39	1.94 (0.36)	2.24 (0.74)	0.50
Hausdorff et al. (2000)	0.91 (0.05)	0.82 (0.06)	−1.59	2.3 (0.1)	4.4 (0.6)	4.83

“*—” indicates not reported*.

## Discussion

The assessment of long-range correlations in the stride-to-stride fluctuations during walking using DFA has become a popular methodology to study movement deficits due to aging as well as degenerative neurological disorders such as PD (Hausdorff et al., 2000; Malatesta et al., 2003; Baltadjieva et al., 2006; Delignieres et al., 2006; Hausdorff, 2007, 2009). In this context, it remains unclear whether the scaling exponent α provided by DFA is able to reflect the health of an individual by determining the nature of long-range correlations within their walking patterns. The main goal of this study was to document the strength of scaling exponent α for investigating age- and pathology-related differences, but also critically review the methodological aspects associated with DFA analysis of human walking data. As such, we aimed to establish current consensus on the application of DFA applied to human walking data and enable improved homogeneity across interstudy comparisons.

Through meta-analysis of the systematically identified review data, our current study has enabled the derivation of optimal thresholds for the scaling exponent α, 0.86 (0.76, 0.96), and 0.82 (0.72, 0.92), which discriminate age- and PD-related influences on the nature of long-range correlations of stride interval of walking. Optimal thresholds allow the cross-fertilization of data from a large number of studies to create a benchmark for the temporal organization of movement variability, against which individual or group performances can be compared. Such thresholds would allow the possible use of the scaling exponent α and the DFA algorithm to complement traditional biomarkers of walking to characterize health, age, and pathology related functional status. Such a framework also allows new reference points (for the first time based on physiological boundaries) to be introduced for the scaling exponent α to replace the traditional theoretically anticipated extreme possibilities of uncorrelated white noise (α = 0.5, understood as pathological) and 1/*f* pink noise (α = 1, understood as healthy), with a clear practical approach for guiding the interpretation of DFA metrics. Examination of group differences revealed more effective identification of PD-related vs. age-related differences using the scaling exponent α, even though the overall ESs appear to be modest in both groups (−0.53 vs. −0.2). However, the observed ESs may have additional practical or clinical value (when interpreted contextually, Durlak, 2009; Gow et al., 2017) due to the metric's non-linearity, and future studies are therefore needed to associate persistence of walking behavior with functionality of the locomotor system (Manor and Lipsitz, 2013; Harrison and Stergiou, 2015; Ducharme et al., 2019). Nevertheless, the modest ESs identified partly highlight issues with relative consistency of scaling exponent α values reported across population groups (confirmed by Cochran's *Q* and *I*^2^ statistics). This is plausibly attributable to the heterogeneity in population demographics of pooled studies. The source of this heterogeneity could be assigned to the severity of disease [the walking patterns of patients with PD with only mild symptoms seem to not deviate considerably from age-matched asymptomatic controls but still show slightly higher α values than patients with advanced PD (Bartsch et al., 2007; Ota et al., 2014)], levels of physical activity [physically active older adults might not differ greatly from healthy young adult performance (Stout et al., 2016; Ducharme et al., 2019)], or fall risk, as well as fear of falling status [older adults who have not experienced a fall vs. those who have fallen previously (Herman et al., 2005; Hausdorff, 2007; Li et al., 2019)]. These issues notwithstanding, a reduction in methodological inconsistencies could contribute to increasing the reliability of DFA analyses. Here, our review highlights that the observed differences in scaling exponent α values across population groups could also be an artifact of methodological differences between studies (see Table 1). As a result, an understanding of how the methodological choices including experimental setup (e.g., duration of walking trial or data series length), choice of input parameters (e.g., range of scales), and environmental constraints (e.g., overground vs. treadmill walking) could affect scaling exponent α need to be achieved.

Given N, the length of a time series, the DFA algorithm fits a power law to the time series' detrended average fluctuations, *F*(*n*), across different window sizes (or scales), *n*, and scaling exponent α is then determined as the slope of log *F*(*n*) vs. log n (refer to Hausdorff et al., 1995; Peng et al., 1995b; Goldberger et al., 2002; Damouras et al., 2010, for methodological details of DFA). Here, DFA has been shown to be dependent on N (a ubiquitous constraint in analyzing most physiological signals) and ensuring reliable estimation of DFA requires at least 500 to 600 strides (Delignieres et al., 2006; Damouras et al., 2010; Almurad and Delignieres, 2016). Although critical, it is generally challenging to acquire such long, continuous, walking datasets, particularly in pathological populations or older adults. Because the required time (generally upward of 15 min; Marmelat and Meidinger, 2019) can lead to fatigue in many clinical populations, support structures such as handrails are often used for safety, which have also been shown to alter DFA (Chang et al., 2009). A feasible alternative here is the use of a safety harness system (without body weight support) that does not influence the scaling exponent α of different spatiotemporal parameters during walking (Stout et al., 2016). Another approach that has been recently tested to mitigate the issue of short data series (but also used traditionally while handling outliers; Hausdorff et al., 1997; Herman et al., 2005; Gow et al., 2017) involves concatenating discontinuous sets of time series (Orter et al., 2019). It has been shown that, for positively correlated signals (1.5 > α > 0.5), such concatenation does not affect the scaling behavior on average (Chen et al., 2002; Gow et al., 2017), but the scaling exponent itself might not be consistent (Kirchner et al., 2014; Marmelat et al., 2018). Bartsch et al. (2007) proposed a modified DFA method to obtain reliable scaling exponent α values in short time series, but such approaches require further investigation, especially in light of a new study showing that scaling exponent α values from shorter walking trials (e.g., 3 min) do not sufficiently capture the fluctuation dynamics of longer time series (Marmelat and Meidinger, 2019). Pursuing this issue in more detail, Kuznetsov and Rhea (2017) proposed a simulation framework to estimate experimental power (number of subjects – number of trials per subject) *a priori*, which shows promise in identifying reliable differences between groups with relatively short (*N* ~ 200) time series of stride interval of walking. In summary, although the aforementioned approaches are encouraging, further studies are needed to systematically test their validity against longer continuous walking trials.

Apart from the data length, it is also expected that the choice of window sizes can affect the estimation of scaling exponent α (Manor and Lipsitz, 2013). This input parameter “n” must be fixed and is generally limited to a range of window sizes, within which the linear relationship between *F*(*n*) and *n* is most stable. Previous studies examining this aspect have recommended the use of 16 to *N*/9 window size (Damouras et al., 2010), but other sizes have also been utilized (Franz et al., 2015; Gow et al., 2017; Marmelat et al., 2018). Another aspect—the sampling frequency of the 3D kinematics data—can also have a considerable impact on scaling exponent α (a lower sampling frequency may reduce the strength of long-range correlations). Here, it has been demonstrated that sampling at ~120 Hz is sufficient to reliably capture the subtle variations in gait cycle duration using scaling exponent α (Liddy et al., 2019). Walking speed may also shape the nature of long-range correlations in walking. In this respect, a reduced strength of long-range correlations at preferred locomotion speeds (quadratic relationship between walking speed and scaling exponent α) might reflect enhanced stability and adaptability (Jordan et al., 2007; Bollens et al., 2012; Chien et al., 2015).

Much of the available evidence regarding the temporal organization of walking variability has focused on time series of stride intervals. While persistence in stride intervals may effectively contain useful characteristics associated with the health of the neural system for regulating rhythmic movements, it is currently unknown if similar characteristics exist in terms of long-range correlations in other spatiotemporal parameters of walking and which aspects of neural deficits they represent. Previous studies have shown that step width (Kaipust et al., 2012; Stout et al., 2016; Franz et al., 2017), stride length (Dingwell and Cusumano, 2010; Roerdink et al., 2015), and even toe clearance (Khandoker et al., 2008) all exhibit persistent long-range correlations. However, perturbations [e.g., walking under environmental constraints such as in a narrow space (Dotov et al., 2016), on a treadmill (Frenkel-Toledo et al., 2005; Warlop et al., 2006; Hollman et al., 2016), paced by a metronome (Roerdink et al., 2015), or while holding handrails (Chang et al., 2009)] have been shown to influence the nature of long-range correlations in these walking parameters. During periodic cued walking (i.e., being paced using, e.g., a metronome), long-range correlations in stride interval of walking have been shown to change from a persistent to antipersistent or random-like temporal organization (Hausdorff et al., 1996; Terrier et al., 2005; Delignieres and Torre, 2009; Kaipust et al., 2013; Marmelat et al., 2014; Roerdink et al., 2015). Similarly, treadmill walking induced antipersistency in stride speed but not in the stride interval and stride length time series (Dingwell and Cusumano, 2010; Terrier and Deriaz, 2012; Roerdink et al., 2015). In this respect, it has become apparent that first principle models, describing the physiological implications for long-range correlations in walking patterns, are needed to elucidate the observed differences between population and parameter groups. Some studies have paralleled the nature of persistence (and its adaptations with aging and pathology) to the level of “tightness” of regulation by neural systems (Dingwell and Cusumano, 2010; Roerdink et al., 2015, 2019). On these grounds, antipersistence in stride speed during treadmill walking is said to reflect “tight” regulation (rapid corrections of any deviations due to internal or external perturbations).

A proper understanding of the relationship between the scaling exponent α during walking and the health of an individual is of paramount importance. Human walking is continuously regulated by central and peripheral neural resources that are involved in the coordination of musculoskeletal systems (Takakusaki, 2017; Ravi et al., 2019). Long-range correlations are conventionally considered to reflect functional interactions among these systems operating at different spatiotemporal scales within the body (Hausdorff et al., 1995, 1997; Goldberger et al., 2002; Herman et al., 2005; Hausdorff, 2007). Long-range correlations have also been recognized as an indicator of the optimal state of motor performance (1/*f* or pink noise, aligned with the theoretical framework of optimal movement variability; Stergiou and Decker, 2011; Harrison and Stergiou, 2015; Cavanaugh et al., 2017), whereas the loss of such correlations with aging and disease is thought to reduce the adaptive capabilities of the individual (a split-belt walking study by Ducharme et al., 2019) confirms these impressions). Within this framework, at one extreme, an absence of long-range correlations (i.e., white noise, α close to 0.5) indicates unconstrained variability and instability in motor performance. At the other extreme (i.e., brown noise, α = 1.5), overly persistent behavior indicates inflexibility and rigidity, reducing the capacity for motor function to flexibly adapt to the demands of the situation (Harrison and Stergiou, 2015). That said, a number of issues also have been raised regarding the application of DFA analyses to human walking. Maraun et al. (2004) argued that the algorithm is highly susceptible to false positives. Similarly, Bryce and colleagues questioned the bias introduced to the estimation of scaling exponents by the algorithm (Bryce and Sprague, 2012). Departing from the original methodology of DFA introduced by Peng et al. (1995b), variants including multifractal DFA (which provides a spectrum of scaling exponents; Kantelhardt et al., 2002; Ihlen, 2012; Ihlen and Vereijken, 2013; Cavanaugh et al., 2017), unbiased DFA, and evenly spaced DFA (lower variability of estimation of scaling exponent α; Almurad and Delignieres, 2016; Yuan et al., 2018) have also recently been introduced. However, in order to gain a deeper understanding of their applicability, it is critical that the compilation of evidence on scaling exponent α across population groups is strictly tied to the particular algorithm used in their estimation.

One aspect of critical importance in the collection of reliable data seems to be adherence to standard methodology. However, the studies presented in this review of the literature exhibit heterogeneity on several key parameters. To facilitate standardization across studies, we therefore present guidelines and recommendations to ensure studies are able to achieve sufficient levels of reliability in the calculation, reporting, and interpretation of scaling exponent α using DFA.

### Recommendations for Calculating, Reporting, and Interpreting Scaling Exponent α Using DFA for Human Walking Data

The following recommendations are derived from the qualitative analysis performed within this systematic review (see *Methodological Choices and Other Study Characteristics*, Table 1) and represent literature-endorsed practices (signified as^†^), as well as empirical evidence. Our aim in reporting these recommendations is to provide comprehensive guidelines for the practical usage of DFA for understanding the fractal dynamics of human walking.

**A. Calculation**

1) ^**†**^**Experimental setup:** In general, investigators should devote sufficient time to understand how certain constraints within their study design (e.g., use of walking aids, harness support etc.) influence the dynamics of human walking and their associated study hypotheses.
2) ^**†**^**Power and sample size estimation:** It is critical that power calculations precede gait variability experiments in order to ensure that DFA scaling exponent α is able to differentiate between cohorts. These calculations should identify the number of subjects and trials required to detect a minimum difference in scaling exponent α ~ 0.10 (2 times the standard error identified in this review) between subjects and trials. Because the number of trials evaluation is usually neglected, it is important in a repeated-measures type of statistical study design to clearly establish if different strategies between subjects exist, which may instead require a single subject analysis.
3) ^**†**^**Resolution of data:** It is recommended that segment kinematics should be collected at ≥120 Hz in order to reliably capture the subtle variations in the dynamics of human walking.
4) **Linear filtering/smoothing of the raw data:** Avoid filtering the kinematic data where possible (or at least only consider with caution) in order to capture the true dynamics of human walking.
5) ^**†**^**Length of time series:** It is recommended that DFA is applied to walking time series of at least 500 to 600 strides captured under continuous and near straight-line walking.
6) ^**†**^**Window sizes (or scales):** Window sizes of 16 to *N*/9 are suggested as the range to calculate the slope of log *F*(*n*) vs. log *n*, where “*N*” is the time series length and “*n*” is the window size.
7) **Removal of outliers from discrete time series:** Use of a median filter is recommended as long as the procedure does not remove data points that reflect the intrinsic dynamics of the system.
8) ^**†**^**Order of DFA detrending:** Typically, a second-order detrending procedure should be employed in the determination of DFA scaling exponent α. Higher-order detrending may be required to eliminate crossovers arising from trends in the data.
9) ^**†**^**Evenly-spaced vs. logarithmically spaced DFA:** To reduce the variability involved in estimating the scaling exponent α, derivation of α should occur from an evenly spaced DFA plot, rather than from a logarithmically spaced DFA plot.
10) **Calculating fit (*R***^**2**^**) of exponent α**: so that the veracity of the exponent can be understood.

**B. Reporting**

11) **Reproducibility:** In addition to the main outcome (mean ± SD of the scaling exponent α), a summary of all the study information necessary to allow a comparative analysis should be readily accessible to the research community. This includes *methodological choices* made in the study (e.g., length of time series, range of window lengths), *population demographics* (severity scores of pathology population, e.g., UPDRS or Hoehn and Yahr scale for subjects with PD, physical activity scores in case of healthy adults), and other potentially *confounding factors* (medications, experimental setup). In regard to the experimental setup, a variety of factors that can influence α should be reported, including clothes and shoes worn in subject testing, surface of support and compliance estimates where possible, ambient noise and temperature conditions, and time of day of testing.

**C. Interpretation**

12) **Optimal movement variability:** Detrended fluctuation analysis evidence of persistent long-range correlations theoretically indicates that α ~ 1.0 signifies an optimal state of adaptability in motor performance, whereas α ~ 0.5 suggests unconstrained variability and unstable motor performance. Here, it remains unclear whether optimal values are task specific. In this respect, further study is required before a full understanding of the optimal values can be obtained.
13) ^**†**^**Screening using optimal thresholds:** A scaling exponent α threshold of 0.86 (CI, 0.76–0.96) and 0.82 (CI, 0.72–0.92) practically discriminates movement performance between age- and PD-related influences regarding the nature of long-range correlations of stride interval of walking.

These recommendations have been collated based on current consensus and empirical evidence and may require revision, enhancement, and/or additions in future as the field of fractal physiology advances.

## Conclusions

The technical and conceptual advances in the methods for analyzing the temporal organization of walking variability have shed light on the complexity of human walking behavior, with DFA methodology playing a key role. However, a general lack of interpretability of both the concept and associated metrics have precluded DFA from providing health-related persistence information on walking. In response to the aims set out in this systematic review and meta-analysis, we have been able to provide evidence that (1) DFA scaling exponent α is only modestly associated with age and PD-related group differences (ES, −0.2 and −0.53, respectively). Here, the DFA metric likely represents a specific characteristic of motor function within the neurophysiological system, thus contributing toward a holistic perspective on individual health. (2) The optimal thresholds for the scaling exponent α = 0.86 (0.76, 0.96) and 0.82 (0.72, 0.92) differentiating age- and pathology-related adaptations, respectively, on walking behavior might now allow a more sensitive and practical application of this metric for understanding temporal regulation of stride interval of walking, and (3) further methodological clarifications regarding DFA would enable the usability of this method in clinical and research settings. In this respect, the reliability of this metric can be enhanced by adhering to the recommendations regarding methodological details provided in this article.

## Data Availability Statement

The raw data supporting the conclusions of this article will be made available by the authors, without undue reservation, to any qualified researcher.

## Author Contributions

DR, NBS, WT, and NS conceived and designed the review. DR and NBS performed literature search, screening, and data extraction. DR, NBS, and WT did the subsequent meta-analyses. VM, NS, and KN provided critical content expertise while drafting and revising the manuscript. KN provided critical opinion and revision of the manuscript as a subject expert. WT is the guarantor. All authors reviewed and approved the manuscript for submission.

## Conflict of Interest

The authors declare that the research was conducted in the absence of any commercial or financial relationships that could be construed as a potential conflict of interest.
