# Supplementary material for: Assessing the Temporal Organization of Walking Variability: A Systematic Review and Consensus Guidelines on Detrended Fluctuation Analysis
Source: Front Physiol. 2020 Jun 23;11:562. doi: 10.3389/fphys.2020.00562 (PMC7324754; doi:10.3389/fphys.2020.00562)
Supplement: Supplementary file 1 [file Data_Sheet_1.PDF]

Supplementary Materials:

Supp. Methods 1. Search String

- (walk* OR gait OR locomot* OR movement OR ambulat*)

- AND (“nonlinear dynamics” OR “stride interval dynamics” OR “stride-to-stride fluctuations” OR variab* OR “Detrended fluctuation analysis” OR “scaling exponent” OR “Fractal dynamics” OR fract* OR “long-range correlation” OR Entropy OR “Sample entropy” OR complexity)

- AND ("Alzheimer disease" OR Dementia OR "Morbus Parkinson" OR Parkin* OR Stroke OR neurology OR "movement disorders" OR "Brain Damage" OR "Brain injuries" OR "Parkinsonian Disorders" OR Multiple schlerosis OR elder* OR old* OR geriatric* OR age OR ageing OR ageing)

- NOT (child* OR animal* OR rats OR monkey* OR mice OR robot* OR amput* OR arm OR hand OR gene OR DNA OR RNA OR "Upper Extremity" OR "Genome Components" OR "Nucleic Acids" OR microbiology OR bacteria OR viruses OR shoulder OR elbow OR insects OR sleep OR "rapid eye movement" OR heart OR cardiac* OR coron* OR pulmon* OR blood* OR diabetes OR hypertension)

Supp. Methods 2. Exclusion criteria

- No information about scaling exponent alpha of stride interval
- Exponents other than alpha calculated using detrended fluctuation analysis
- Case studies
- Studies that did not adhere to the general operational definitions of gait parameters (with respect to the choice of equipment)
- Neurological pathologies other than Parkinson's disease
- No comparison against healthy control group
- Studies with non-representative samples (athletes, children, amputees)
- No walking task
- Walking speed other than self-selected
- Only perturbed/metronome walking task
- Daily life gait/outdoor walking/ walking on virtual environments
- Ramp ascent and descent tasks
- Walking with aids/body weight supported treadmill walking
- Attention demanding task added (dual task conditions)

Fig. S1. Binary Logistic Regression plot for Young Adults vs Older Adults. (red- pathological, green - healthy)

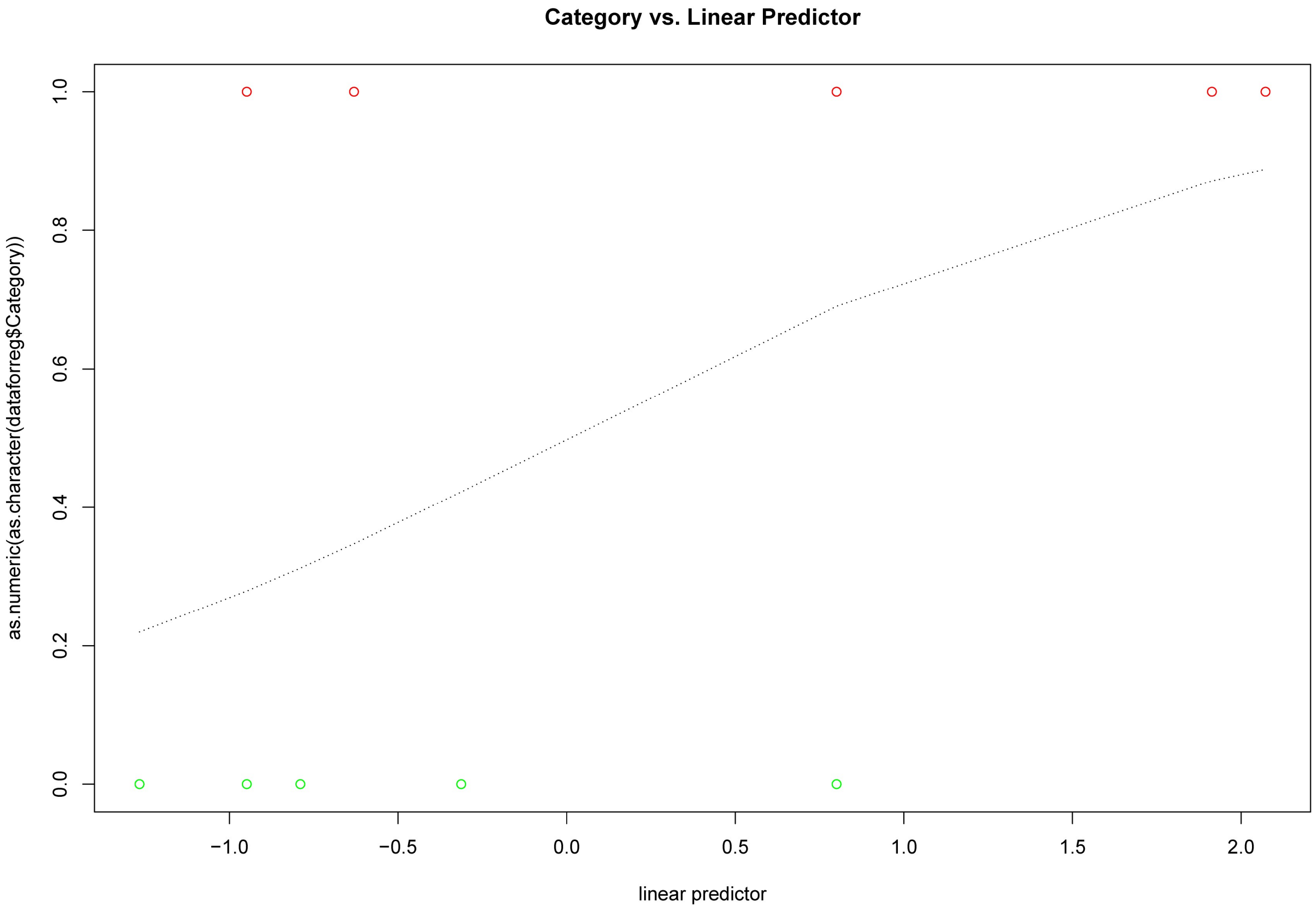

Fig. S2. Receiver operating characteristic curve for Young Adults vs Older Adults

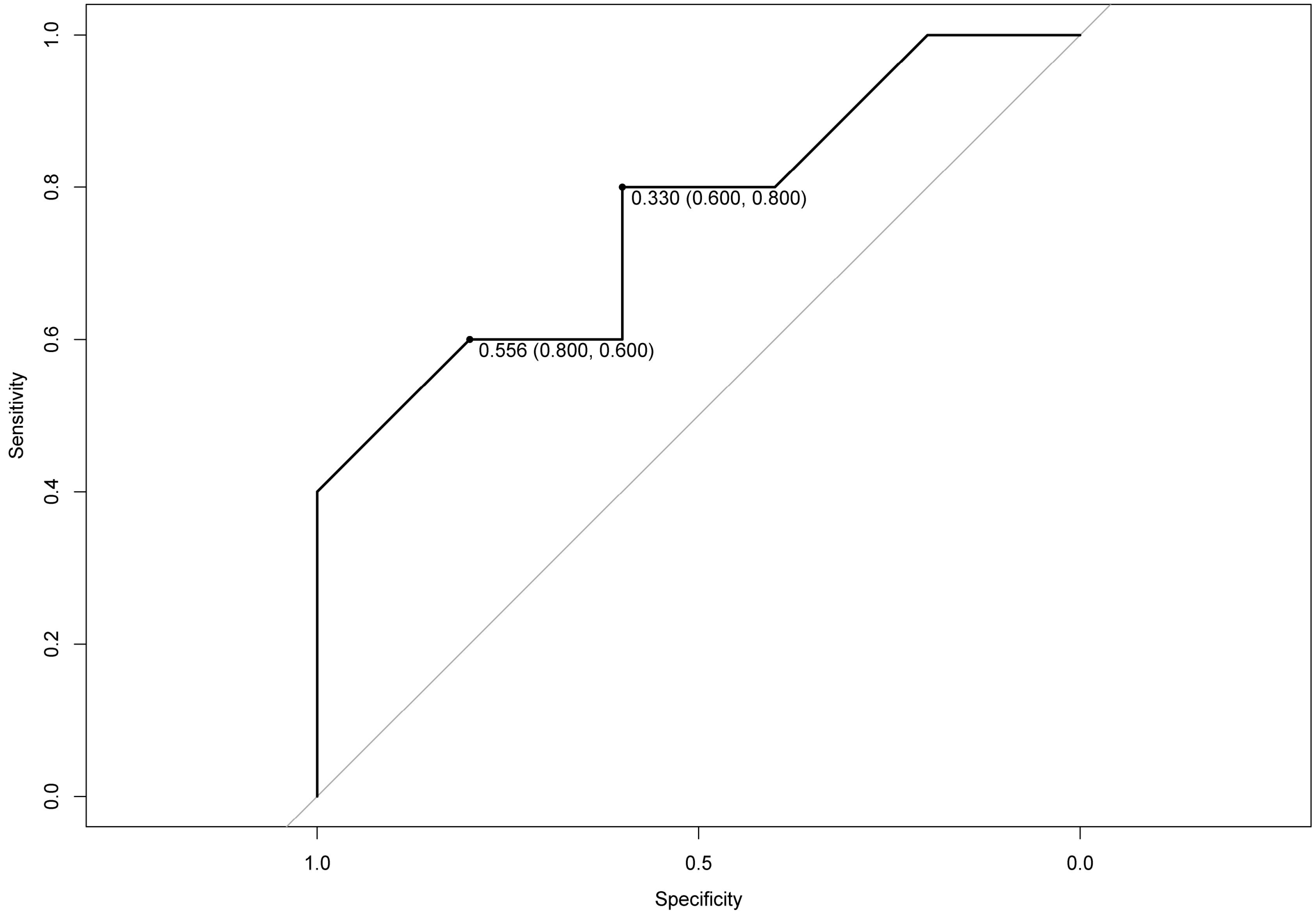

Fig. S3. Binary Logistic Regression plot for Patients with Parkinson's disease vs Matched Healthy Controls. (red- pathological, green - healthy)

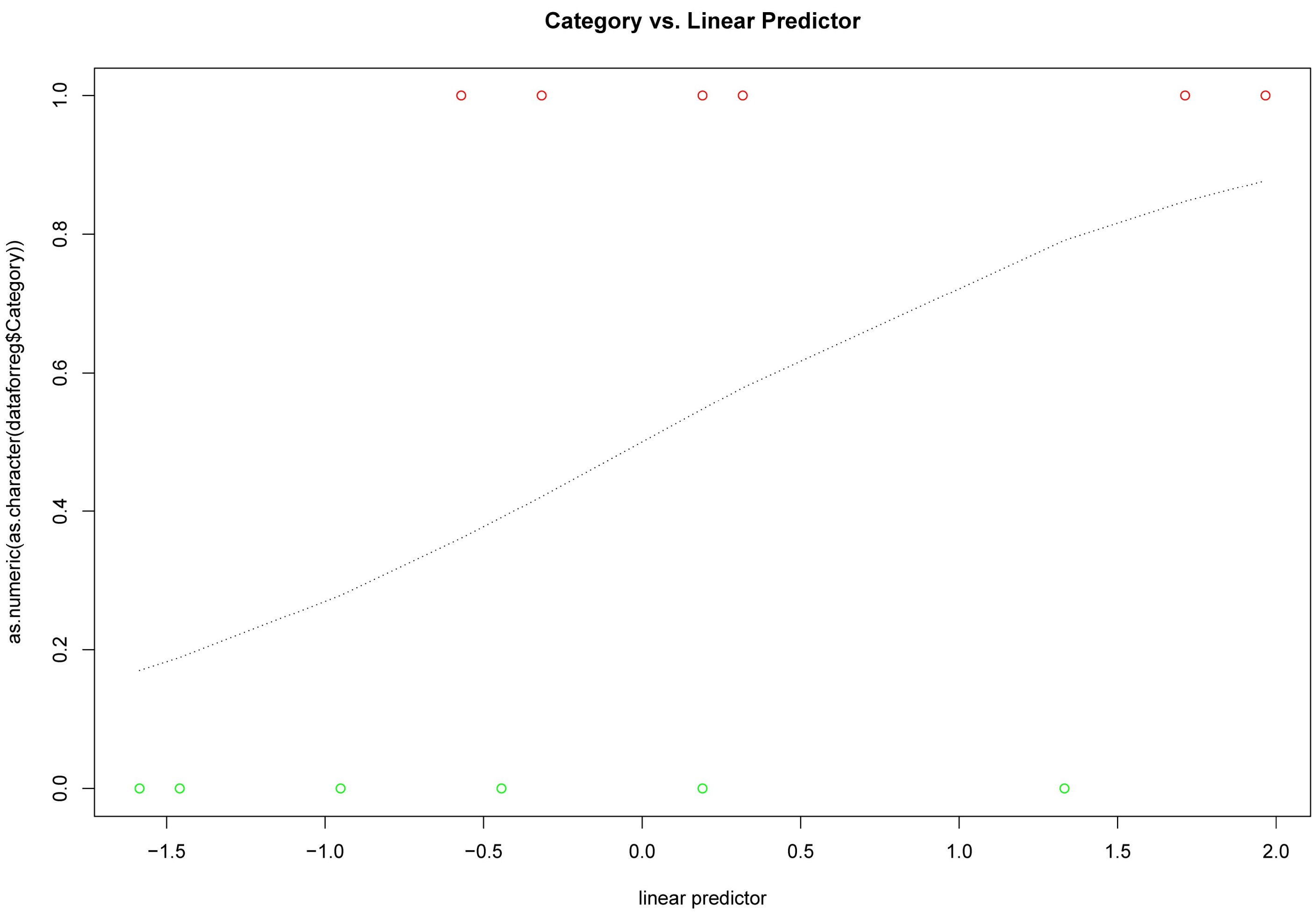

Fig. S4. Receiver operating characteristic curve for Patients with Parkinson’s disease vs Matched Healthy Controls

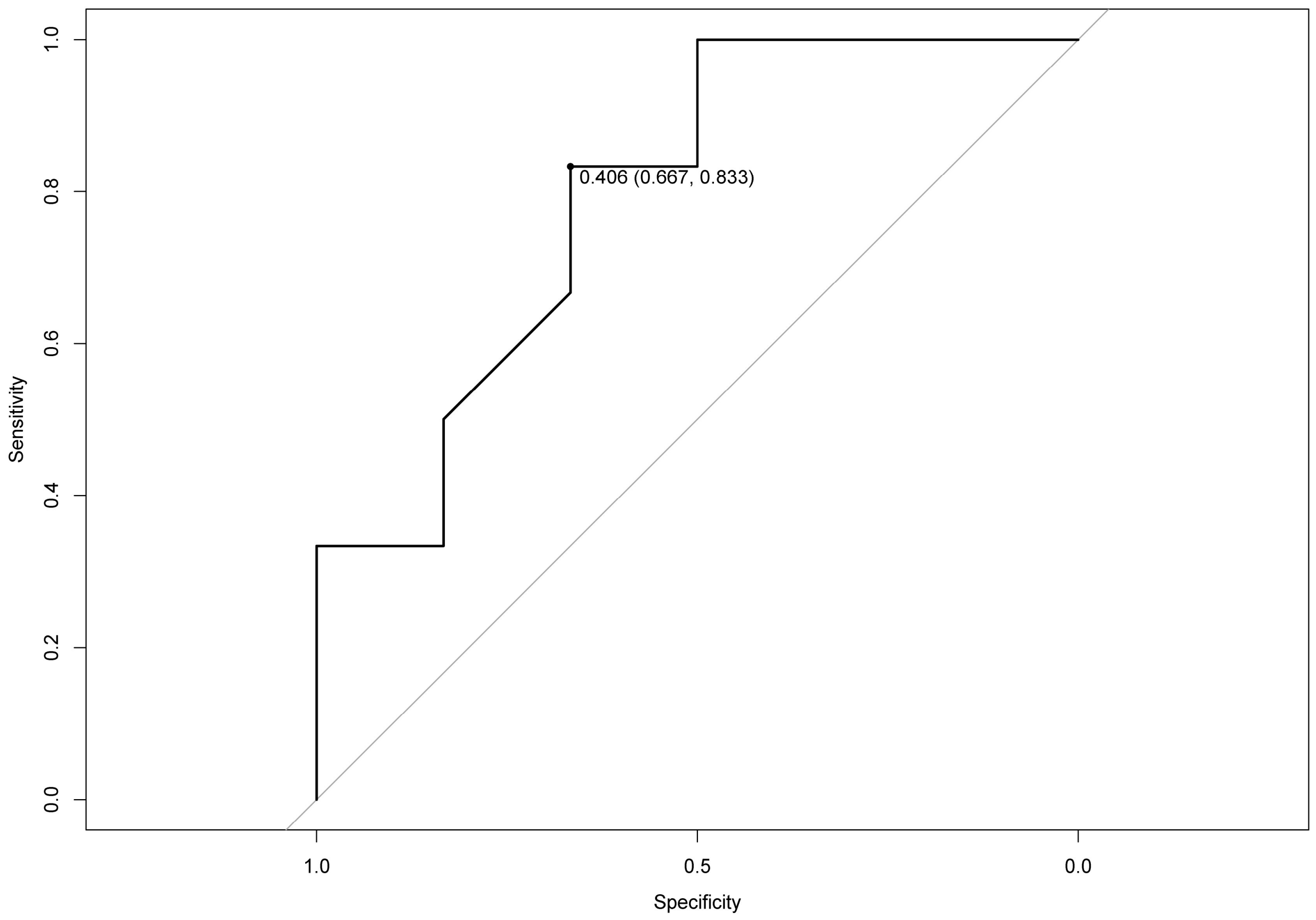

Table S1. Quality assessment of studies included in the review

Author (Year)	Clearly Stated Aim	Inclusion of Consecutive Patients	Prospective Collection of Data	Endpoints appropriate to the aim of the study	Prospective calculation of the study size	An adequate control group	Contemporary groups	Baseline equivalence of groups	Adequate statistical analyses	Total
**Marmelat (2018)**	2	2	2	2	0	1	2	2	2	15
**Dotov (2017)**	1	2	2	2	0	1	2	1	2	13
**Stout (2016)**	2	2	2	2	0	1	2	1	1	13
**Kosse (2016)**	2	2	2	2	0	0	0	1	2	11
**Chien (2015)**	2	2	2	2	0	1	2	1	1	13
**Ota (2014)**	2	2	2	2	0	0	2	1	2	13
**Kobsar (2014)**	2	2	2	2	0	1	2	2	1	14
**Kaipust (2013)**	2	2	2	2	0	1	2	1	2	14
**Bartsch (2007)**	1	1	2	2	0	1	2	1	1	11
**Warlop (2006)**	2	2	2	2	0	1	2	2	2	15
**Toledo (2005)**	2	2	2	2	0	1	2	1	1	13
**Malatesta (2003)**	2	2	2	2	0	1	2	2	1	14
**Hausdorff (2000)**	2	1	2	2	0	1	0	1	2	11
**Hausdorff (1997)**	1	2	2	2	0	1	2	2	1	13

Scored as 2 (low risk), 1 (unclear risk), 0 (high risk)
